# Sensor based sleep patterns and reported sleep quality in breast cancer patients undergoing neoadjuvant chemotherapy

**DOI:** 10.1038/s41598-025-99208-0

**Published:** 2025-07-11

**Authors:** Carla Malveiro, Sofia Boavida, Catarina Cargaleiro, Ana V. Bernardino, Inês R. Correia, Cátia Reis, Leonor Matos, Luís B. Sardinha, Maria João Cardoso, Pedro F. Saint-Maurice

**Affiliations:** 1 Champalimaud Foundation, Lisbon, Portugal; 2 Exercise and Health Laboratory, CIPER, Faculdade de Motricidade Humana, Universidade de Lisboa, Cruz-Quebrada, Portugal; 3 CRC-W - Católica Research Center for Psychological, Family and Social Wellbeing, Faculdade de Ciências Humanas, Universidade Católica, Lisbon, Portugal; 4 GIMM - Gulbenkian Institute of Molecular Medicine, Lisbon, Portugal; 5 ISAMB - Instituto de Saúde Ambiental, Faculdade de Medicina, Universidade de Lisboa, Lisbon, Portugal; 6 Breast Unit, Champalimaud Foundation, Lisbon, Portugal; 7 Faculty of Medicine Science, Nova University, Lisbon, Portugal

**Keywords:** Breast cancer, Neoadjuvant chemotherapy, Sleep patterns, Insomnia, Quality of life, Health care, Oncology

## Abstract

Breast cancer is the most diagnosed cancer in women worldwide and its treatment often leads to the onset of sleep disturbances. While much research has focused on chemotherapy’s impact on overall sleep quality through subjective measures, less attention has been given to its effects on specific sleep metrics such as duration, timing, continuity, and naps. This preliminary study addresses this gap by assessing sleep duration, timing, and regularity, using the Emfit QS device over 100 consecutive days in 24 breast cancer patients undergoing neoadjuvant chemotherapy. Additionally, we incorporated the Pittsburgh Sleep Quality Index (PSQI) to measure reported sleep quality. Our results suggest that chemotherapy may influence the duration for time spent in bed (ptrend = 0.02) measured by the Emfit QS. Duration in bed decreased over the first seven weeks (e.g., 9.3 h/day at week 1 vs. 8.5 h/day at week 8), and increased thereafter to similar amounts as those recorded in week 1 (9.0 h/day at week 15). Sleep timing and regularity, also measured by the Emfit QS, remained unchanged. Overall sleep quality, as measured by the PSQI, did not change over time. However, our analysis of the individual components of the PSQI revealed that sleep disturbances increased as treatment progressed from week 1 to week 8 (1.3 ± 0.6 to 1.7 ± 0.6; *p* = 0.01), concurrently with an increase in insomnia symptoms. Approximately, 33%, 63%, and 73% reported having insomnia symptoms at week 1, 8, and 15. These findings highlight critical periods during treatment when patients are vulnerable to disrupted sleep. Future research should focus on interventions to mitigate sleep disturbances, improving patient well-being and overall quality of life.

**Supplementary Information:**

The online version contains supplementary material available at 10.1038/s41598-025-99208-0.

## Introduction

Breast cancer is the most frequently diagnosed cancer in women worldwide making up 11.6% of all cancers^1^. Breast cancer treatment frequently requires neoadjuvant chemotherapy that can lead to severe side effects such as nausea, fatigue, and pain^2^. These side effects can promote circadian disruption, i.e., mistiming of sleep and physiological processes, and inconsistent sleep patterns, and have a subsequent impact on patients’ quality of life and prognosis^3–5^. The effect of neoadjuvant chemotherapy on sleep quality is well studied but whether treatment impacts other dimensions of sleep including sleep duration, timing, and regularity, remains largely unknown^6^. Understanding how chemotherapy influences sleep dimensions is essential for developing effective sleep interventions and potentially improve the quality of life and prognosis of women diagnosed with breast cancer.

Sleep disorders affect 67–90% of women diagnosed with breast cancer^7^, and sleep disruption is particularly exacerbated during active treatment. Women undergoing treatment for breast cancer often experience poorer sleep quality when compared to women not receiving treatment (prevalence: 70% vs. ~58%, respectively)^8^. Sleep patterns tend to deteriorate significantly by the tenth week of chemotherapy with reports of shorter sleep durations and poorer sleep quality relative to pre-treatment sleep patterns^9^. The prevalence of sleep disorders, including insomnia, is also particularly high among breast cancer patients during treatment^10–12^. However, the evidence clarifying whether sleep patterns are disrupted as a result of treatment for breast cancer remains incomplete.

Most studies of sleep during cancer treatment have used subjective measures of sleep (e.g., questionnaires)^13^ that can offer valuable insights for sleep quality but that have limited accuracy for quantifying dimensions of sleep related to duration, timing, and regularity^14^. Device-based assessments using actigraphy can improve the accuracy of sleep measurements overall but implementing actigraphy over extended monitoring periods (i.e., months) can be challenging^15^. Contactless and sensor based sleep devices are becoming more prominent since this technology can collect sleep information passively and for long periods of time without disrupting cancer patients sleep routines^15–18^. Studies of chemotherapy treatment have also focused on sleep quality^9,19^ and have not examined sleep timing or day-to-day sleep variation/regularity, an important marker for circadian disruption^20–22^. Therefore, the extent that treatment impacts the various dimensions of sleep during breast cancer treatment remains unclear, though clarifying this could inform future sleep interventions targeting sleep disruptions in cancer patients.

Our study fills an important gap by characterizing sleep patterns related to duration, continuity, timing, regularity, and naps, among breast cancer patients undergoing neoadjuvant chemotherapy. In this study we leveraged sensor based sleep technology to obtain more objective characterizations of sleep throughout the entire duration of chemotherapy treatment, i.e.,120 consecutive days or 4 months.

## Methods

### Patients and study design

This is a preliminary study conducted as part of a 4 to 6 month randomized controlled trial examining the impact of exercise on Ki67 among breast cancer patients undergoing neoadjuvant treatment (The Neoadjuvant Exercise Oncology Program – NEO -Program: ClinicalTrials.gov Identifier: NCT05297773. First Posted: 28-03-2022) (Supplement S1).

The current study includes 28 women newly diagnosed with breast cancer who accepted to participate in a 4 to 6 month sleep measurement protocol. This study was approved by the Champalimaud Foundation Ethics Committee and patients were required to provide signed consent to participate. To be eligible to the study women had to be at least 18 years old, diagnosed with hormone-receptor positive/HER2-negative breast cancer (stage 0 to III), scheduled to receive neoadjuvant chemotherapy, and not have participated in structured exercise programs in the last 6 months. As part of the overall NEO trial, women also had to be willing to participate in exercise sessions upon confirmed medical clearance. Women were excluded if they had received cancer treatment (except basal or cervical) in the past 5 years, had uncontrolled heart disease, diabetes, chronic lung diseases, psychological disorders (e.g., dementia, Alzheimer’s, Parkinson’s), severe disabilities limiting exercise, or reported alcohol/drug abuse.

Patients completed a battery of demographic/health-related questionnaires at the beginning of treatment (week 1) and started a sleep measurement protocol that included using a contactless sleep device at home for the entire duration of treatment, which consisted of eight cycles of neoadjuvant chemotherapy (four cycles each of anthracycline-based and taxane-based agents). The dosing and frequency of chemotherapy treatment were individually adjusted ranging from a minimum of 7 to a maximum of 16 cycles. Each cycle involved an infusion of chemotherapy drugs, followed by a recovery phase lasting 1 to 2 weeks. On average each participant had 4.1 ± 0.5 cycles of anthracyclines and 4.4 ± 1.7 cycles of taxanes (8.5 ± 1.9 cycles total). As part of the sleep assessment protocol, patients were also asked to complete a sleep questionnaire at the beginning, mid, and end of treatment. Preliminary analysis with the final sample included in this study showed that the exercise intervention did not impact sleep patterns (*p* = 0.31).

### Measures

#### Demographic characteristics

Demographic information including age, menopause status, education, socioeconomic level, marital status, ethnicity and disease-related information, including tumor subtype and stage were collected using the Research Electronic Data Capture (REDCap) platform. Assessments also included measurements of height, weight, body mass index (BMI), percent body fat using bioelectrical impedance, and maximal oxygen uptake (VO2_max_), which was estimated through a maximal exercise test on a bicycle ergometer.

#### Measurement of sleep

Sleep was measured using the sensor based Emfit QS device. The Emfit is a bed movement sensor (Emfit QS Corp., Kuopio, Finland) that is placed beneath the mattress at the chest level. This sensor is composed of thin elastic light-weight polymer layers divided by air spaces and covered with electrically conductive polarized layers^18^. Each Emfit device has a Subscriber Identification Module (SIM) card that transmitted the data to a protected server. The Emfit uses ballistic movement and proprietary algorithms to estimate sleep patterns and has acceptable accuracy for total time in bed, sleep duration, bedtime, and get up times^15,16^. Epoch-by-epoch sleep/wake classifications between Emfit and actigraphy show moderate agreement (kappa = 0.6), with Emfit exhibiting strong correlations (*r* > 0.9) for timing estimates^15^. Emfit’s performance for detecting awake episodes versus actigraphy reveals a sensitivity of 0.62, specificity of 0.93, and accuracy of 0.88^15^. When compared to polysomnography (PSG), Emfit shows moderate correlations for total sleep time (*r* = 0.5) and time in bed (*r* = 0.64)^17^. Sleep/wake classifications derived from Emfit’s sleep stage timeseries within the lights-off period demonstrate high sensitivity (0.99) but low specificity (0.12), with an overall accuracy of 0.71 compared to PSG^17^. Emfit’s estimation of wake after sleep onset (WASO) correlates poorly with PSG (*r* = 0.26)^17^. These findings suggest that Emfit may serve as a reliable alternative to actigraphy and PSG for measuring sleep timing and duration, although, and similarly to actigraphy, caution is warranted when assessing parameters such as WASO^15–17^. Patients were provided with the Emfit device along with installation instructions and a demonstration video. Patients were instructed to conduct all sleep events, including naps, in their primary bed location where the device was installed.

Subjective sleep quality was obtained using the Pittsburgh Sleep Quality Index (PSQI) at three points: beginning of treatment (T1—week 1), mid-treatment (T2—week 8), and after the final treatment (T3—week 16). The PSQI questionnaire measures sleep quality over the previous month through nineteen self-reported questions, divided into seven categories: subjective sleep quality, sleep latency, sleep duration, habitual sleep efficiency, sleep disturbances, use of sleeping medications, and daytime dysfunction. The PSQI was scored using recommended scoring guidelines, where each category is rated on a 0 to 3 scale. The total score ranges from 0 to 21, with higher scores indicating poorer sleep quality^23^.

### Processing emfit sleep data

The Emfit device continuously monitors sleep data at one-minute intervals using proprietary algorithms. Emfit data was exported in one-minute epochs and aggregated at the day-level. In total, there were approximately 2880 individual days with recorded sleep data. In instances where more than one sleep event was recorded on a given day, the longest sleep event was used as the primary sleep event, while secondary sleep events recorded on the same day were coded as naps. Some patients had more than one nap per day, so multiple naps were averaged to obtain a single nap estimate for each day. We used Emfit algorithms to compute sleep measures related to duration (24 h total time in bed, time in bed for the primary sleep event, sleep duration), continuity (i.e., wake after sleep onset [WASO]), sleep efficiency), and timing (bedtime, get out of bed, and sleep midpoint), and regularity (standard deviation of sleep midpoint). We also extracted information about naps to examine the prevalence of patients with 1 or more naps per day, the time in bed related to naps, and sleep duration during naps. More detailed definitions of the sleep metrics used in our study are provided in Supplement S2. Emfit sleep processed data were then merged with the start and end dates for neoadjuvant chemotherapy, and days recorded outside the treatment window were excluded from further processing.

Seven-day averages were computed over valid days of recorded sleep, starting from the treatment start date. A week of sleep data was considered valid if it included at least one day with recorded sleep. Week averages based on fewer than 10 observations (i.e., patients) were considered suboptimal representations of weekly sleep patterns and were excluded. Patients were excluded if they did not have valid sleep data on at least 75% of the total weeks prescribed for chemotherapy treatment. On average, sleep data were obtained for 15 out of 17 weeks allocated for neoadjuvant chemotherapy (~ 90% of the total treatment period).

### Statistical analysis

Descriptive statistics (mean ± standard deviation) were computed for all measures collected at the beginning of treatment (week1). The main analysis examined the effect of neoadjuvant chemotherapy on sleep patterns over valid weeks of sleep data recorded by the Emfit and reported on the PSQI. We used linear mixed models to fit each sleep indicator against an effect for time since treatment (i.e., week after the first chemotherapy session). Our models included a random effect for patients and an unstructured covariance matrix to account for the nature of repeated observations. Time since treatment was included in the mixed models as a linear (i.e., β_linear_) and quadratic function (i.e., β_quadratic_) to capture non-linear trends in sleep outcomes over the course of treatment. For each sleep outcome, p_trend_ was defined as: (1) the p-value for the linear effect of time if the quadratic term for time was not statistically significant, or (2) the p-value for the non-linear effect of time if the quadratic term for time was statistically significant.

We also used linear mixed models and respective p-values to determine whether PSQI scores differed across the three time points (T1, T2 e T3, i.e., β coefficient for overall time differences). Our secondary analysis examined whether sleep measures differed between weeks with no treatment vs. anthracyclines vs. taxanes. These examinations were conducted using linear mixed models and by modelling each individual sleep metric against a factor with three levels for treatment (no treatment, anthracyclines, taxanes).

As a sensitivity analysis, we conducted additional examinations to assess whether sleep medication and measurement error impacted our results. The main analyses for time in bed were replicated by (1) excluding patients who were not on sleep medication by the end of the trial, and (2) excluding patients who had < 3 days per week of valid sleep data.

All analyses were two-sided, with statistical significance defined as *p* ≤ 0.05, and were conducted using SAS v9.4.

## Results

### Participant characteristics

Of the total of 28 patients who consented to participate in the study, 4 were not compliant with the Emfit sleep protocol, resulting in a final sample of 24 patients: 7 in the Aerobic Training group, 10 in the Strength Training group, and 7 in the Control group. While participants were assigned to groups, all analyses were conducted on the total sample rather than by intervention group. No serious adverse events or unintended effects related to the exercise intervention were reported. Patients were on average 51.9 ± 9.4 years, with a BMI of 25.4 ± 3.6, and all patients were Caucasian. The majority of patients had a diagnosis of breast cancer luminal B-like subtype, had a college degree, were married, and were not on sleep medication at the start of the treatment (Table 1).

**Table 1:** Descriptive characteristics (*n* = 24).

	Mean ± Std
Age (years)	51.9 ± 9.4
Height (cm)	161.2 ± 7.2
Weight (kg)	65.8 ± 8.5
BMI (kg/m^2^)	25.4 ± 3.6
Fat mass (%)	35.4 ± 9.0
VO_2max_ (ml/kg/min)	22.7 ± 4.5

	*N* (%)
Tumor subtype	
Luminal A-like	7 (29.2)
Luminal B-like	13 (54.1)
Missing	4 (16.7)
Tumor stage (AJCC 10th Edition)	
Stage II	11 (45.8)
Stage III	9 (37.5)
Missing	4 (16.7)
Menopause status	
Pre-menopausal	9 (37.5)
Post-menopausal	8 (33.3)
Missing	7 (29.2)
Education level^a^	
High School or lower	2 (8.4)
College Degree	16 (66.6)
Missing	6 (25.0)
Socioeconomic status (household €/month)	
1000–1499	3 (12.5)
1500–1999	2 (8.3)
2000–2499	3 (12.5)
> 2500	10 (41.7)
Missing	6 (25.0)
Marital status	
Single	1 (4.2)
Married	17 (70.8)
Divorced	3 (12.5)
Other^b^	2 (8.3)
Missing	1 (4.2)
Sleep Medication (times/week)^c^	
None	18 (75.0)
< 1	1 (4.2)
1 to 2	1 (4.2)
3+	4 (16.6)

^a^Education level: High School or lower, includes 4th and 12th grades; College Degree, includes bachelor andmaster.

^b^Other variable include non-marital partnership and widower status.

^c^Sleep medication is derived from a question in the Pittsburgh Sleep Quality Index.

Patients completed on average 14.8 weeks of neoadjuvant chemotherapy and during this time spent on average 8.9 ± 1.4 h per night in bed, 7.9 ± 1.3 h asleep, and accumulated 1.0 ± 0.4 h of wake after sleep onset (WASO) per night throughout the treatment period. On average, patients went to bed at 23:36, woke up at 8:30, and the sleep midpoint occurred at 4:06 ± 1.3 h. Data from the Emfit on naps showed that 23.7% of the patients took one or more naps per day and among those who napped, they spent an average 3.5 ± 1.2 h in bed, and 2.1 ± 0.8 asleep.

### Sleep duration

**Fig. 1:**
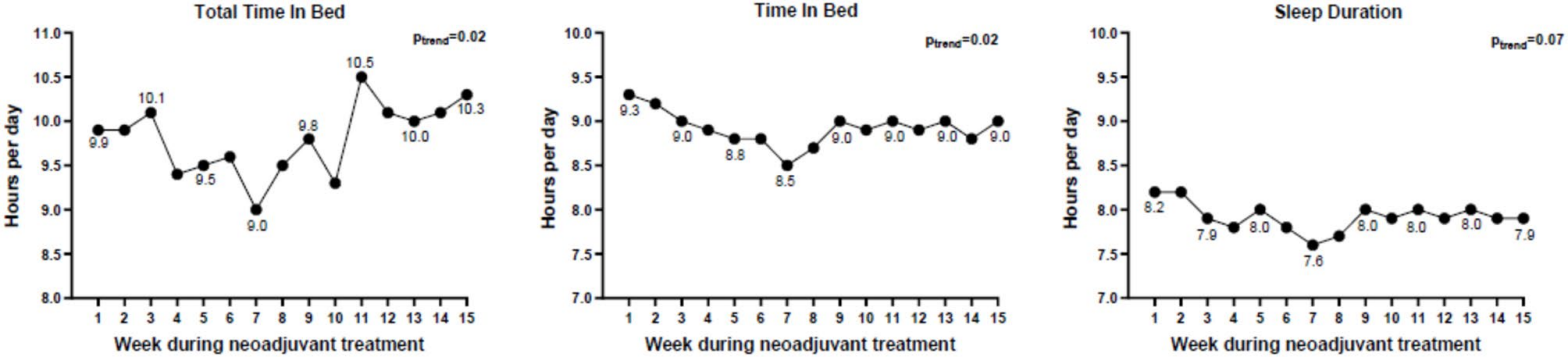
Total time in bed (left), time in bed (middle), and sleep duration (right) over 15 weeks of recorded sleep data. Note: Total time in bed is measured over a 24-hour cycle; Time in bed refers to the duration of the primary/longest sleep event; Sleep duration refers to the time spent sleeping during the primary sleep event.

Our examinations of sleep duration metrics (Fig. 1) revealed that the total time in bed during neoadjuvant chemotherapy exhibited fluctuations over the 15-week period, ranging from 9.0 to 10.5 h per day. Total time in bed followed a curvilinear trend throughout the treatment (β_linear_= − 0.18 ± 0.09; β_quadratic_ = 0.01 ± 0.0; p_trend_=0.02), showing a clear decrease until week 7 of neoadjuvant chemotherapy, followed by a slight increase through week 11 and then stabilizing toward the end of treatment. Similarly, time in bed followed a curvilinear pattern (β_linear_= − 0.12 ± 0.05; β_quadratic_ = 0.01 ± 0.0; p_trend_= 0.02), decreasing until week 7 and then plateauing thereafter. For example, time in bed at week 1, 8, and 15 was 9.3, 8.5, and 9.0 h per night, respectively. Sleep duration followed a similar trend, but results were not statistically significant (β_linear_= − 0.09 ± 0.05; β_quadratic_ = 0.01 ± 0.00; p_trend_=0.07).

### Sleep continuity

**Fig. 2:**
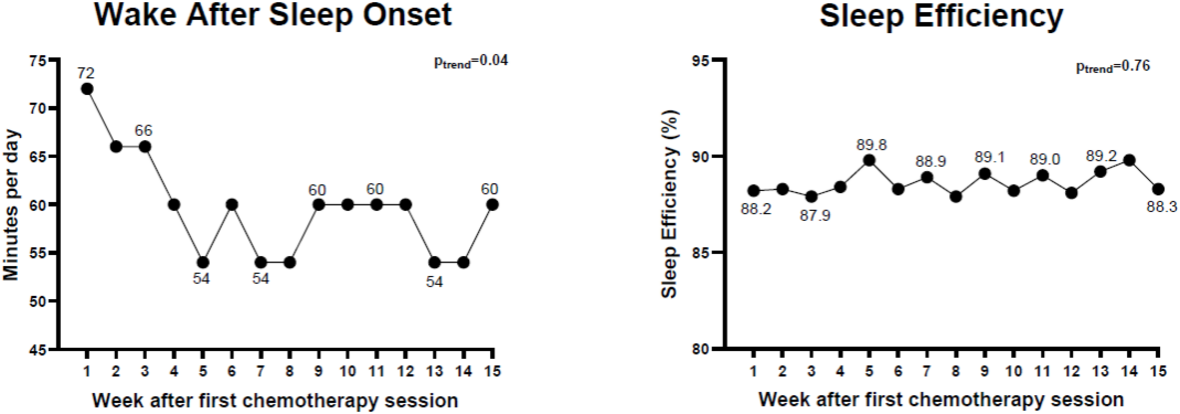
Wake after sleep onset (left) and sleep efficiency (right) over 15 weeks of recorded sleep data. Notes: Wake after sleep onset refers to the amount of time a person spends awake after initially falling asleep, throughout the main sleep period (minutes per day); Sleep efficiency refers to the relationship between sleep duration and time in bed.

The examinations for sleep continuity and sleep efficiency showed that WASO had a curvilinear trend over the duration of neoadjuvant chemotherapy treatment (Fig. 2; β_linear_= − 0.04 ± 0.02; β_quadratic_ = 0.00 ± 0.00; p_trend_= 0.04). Patients spent on average 72, 54, and 60 min per night awake at week 1, 8, and 15 of the treatment, respectively. The ratio of WASO to total time in bed and defined as sleep efficiency did not show any clear trend over time during treatment (β_linear_ = 0.06 ± 0.20; β_quadratic_ = 0.00 ± 0.01; p_trend_= 0.76).

### Sleep timing

**Fig. 3:**
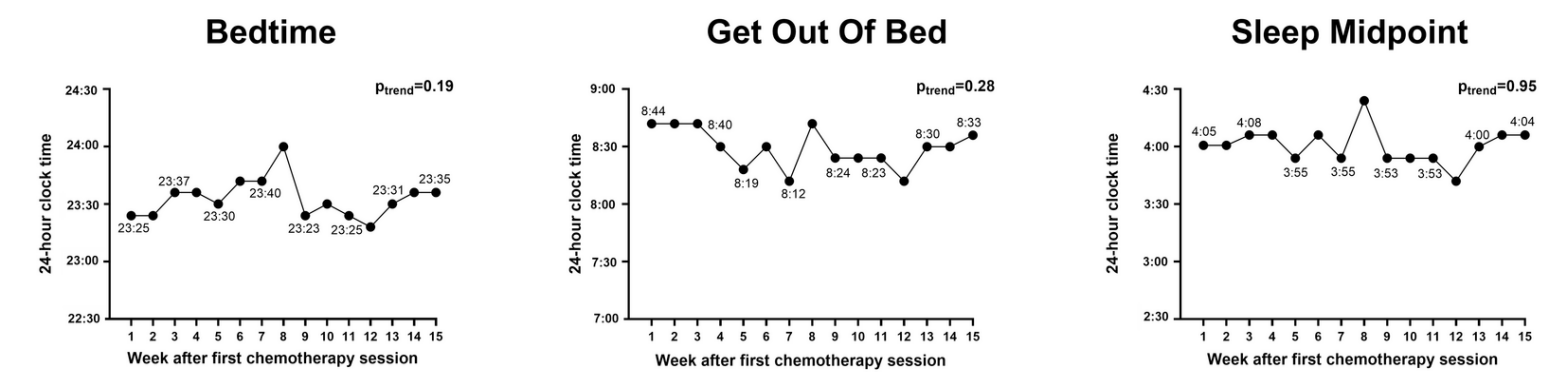
Bedtime (left), get out of bed (middle), and sleep midpoint (right) over 15 weeks of recorded sleep data. Notes: Bedtime refers to the time a person goes to bed; Get out of bed refers to the time a person gets out of bed after waking up, marking the end of their sleep episode; Sleep *m*idpoint refers to the midpoint between bedtime and get up time.

There were no statistically significant trends for bedtime (β_linear_ = 0.06 ± 0.05; β_quadratic_= − 0.00 ± 0.00; p_trend_= 0.19), get out of bed (β_linear_= − 0.06 ± 0.20; β_quadratic_ = 0.00 ± 0.01; p_trend_= 0.28), or sleep midpoint (β_linear_ = 0.00 ± 0.04; β_quadratic_= − 0.00 ± 0.00; p_trend_= 0.95) throughout the duration of treatment (Fig. 3). Sleep regularity also remained stable throughout the duration of treatment (β_linear_= − 0.24 ± 0.24; β_quadratic_ = 0.01 ± 0.01; p_trend_= 0.30) (Supplement S3).

### Naps

The percentage of patients with one or more naps recorded per day did not change significantly throughout treatment (β_linear_= − 0.02 ± 0.02; β_quadratic_ = 0.00 ± 0.00; p_trend_= 0.31). Patients’ time spent in bed during naps ranged from 1.4 to 4.2 h per day during treatment and there was no clear trend throughout the duration of treatment (β_linear_= − 0.05 ± 0.07; β_quadratic_ = 0.01 ± 0.00; p_trend_= 0.47). Similarly, sleep duration during naps ranged from 1.7 to 2.9 h per day, also showing no clear trend over the course of treatment (β_linear_= − 0.02 ± 0.09; β_quadratic_ = 0.00 ± 0.01; p_trend_= 0.84) (Supplement S4).

### Sleep quality

Subjective sleep quality as indicated by total scores reported in the PSQI questionnaire did not change during treatment (Table 2). Scores for PSQI were 7.1, 7.0, and 7.5 for the beginning, middle, and end of the treatment (β = 0.19 ± 0.33; *p* = 0.58). There were no clear differences for PSQI subscales except for sleep disturbances. Patients reported more sleep disturbances as the chemotherapy treatment progressed (*p* = 0.01). Our examination of individual PSQI items for the sleep disturbances subscale showed that patients woke up in the middle of the night or early morning more frequently as the treatment progressed (*p* < 0.01). The proportion of patients that woke up in the middle of night or early in the morning on three or more times per week at beginning, middle, and end of treatment were 33%, 63% and 73%, respectively. Scores for feeling too cold or having bad dreams also increased throughout treatment (*p* ≤ 0.05).

**Table 2:** Pittsburgh sleep quality index at week 1, 8 and 16 of treatment (*n* = 24).

	T1 (*n* = 24)	T2 (*n* = 24)	T3 (*n* = 24)	*p*-value	
	Mean ± Std	Mean ± Std	Mean ± Std		
Subjective sleep quality (0–3)	1.2 ± 0.8	1.2 ± 0.8	1.4 ± 0.9	0.14	
Sleep latency (0–3)	1.3 ± 1.0	1.2 ± 1.0	1.1 ± 1.2	0.40	
Sleep duration (0–3)	0.8 ± 1.1	0.5 ± 0.9	0.8 ± 1.4	0.78	
Habitual sleep efficiency (0–3)	0.9 ± 1.3	0.8 ± 0.8	0.8 ± 1.1	0.75	
Sleep disturbances (0–3)	1.3 ± 0.6	1.4 ± 0.7	1.7 ± 0.6	0.01	
Use of sleep medication (0–3)	0.6 ± 1.2	1.0 ± 1.5	0.8 ± 1.3	0.43	
Daytime dysfunction (0–3)	1.0 ± 0.8	1.1 ± 0.9	0.8 ± 0.8	0.33	
Total score (0–21)	7.1 ± 3.3	7.0 ± 3.2	7.5 ± 3.4	0.58	

T1, week 1 assessment; T2, week 8 assessment; T3, week 16 assessment.

Scale for each variable: Each domain has a scale from 0 to 3, except for the Total Score, which ranges from 0 to 21.

Direction of scores: For all variables, higher scores indicate poorer sleep quality.

### Sleep metrics by type of chemotherapy

Sleep metrics recorded by the Emfit remained similar on weeks with vs. without treatment (Table 3) and there were no clear differences in sleep parameters during anthracyclines vs. taxanes-chemotherapy treatment.

**Table 3:** Sleep metrics for weeks with no treatment vs. anthracyclines vs. taxanes.

	No treatment	Anthracyclines	Taxanes	*p*-value
	Mean ± Std	Mean ± Std	Mean ± Std	
Duration (hrs/d)				
Total time in bed	9.7 ± 2.3	9.6 ± 2.2	10.1 ± 2.5	0.12
Time in bed	8.9 ± 1.4	8.9 ± 1.4	9.0 ± 1.4	0.51
Sleep duration	7.9 ± 1.3	7.9 ± 1.3	8.0 ± 1.3	0.48
Continuity				
WASO (min) Sleep Efficiency (%)	1.0 ± 0.5 88.5 ± 3.4	1.0 ± 0.5 88.8 ± 4.7	1.0 ± 0.4 88.8 ± 4.2	0.75 0.62
Timing (hh: mm)				
Bedtime	23:58 ± 1.6	24:04 ± 1.7	23:44 ± 1.4	0.10
Get out of bed	8:47 ± 1.5	8:51 ± 1.5	8:44 ± 1.4	0.34
Sleep midpoint	4:03 ± 1.4	4:08 ± 1.4	4:34 ± 1.2	0.12
Sleep regularity (Std)	4:12 ± 2.5	4:44 ± 4.8	4:59 ± 5.8	0.30
Naps				
Patients with 1 + nap per day (%)	11.1 ± 8.3	6.0 ± 9.4	7.8 ± 11.4	0.37
Time in bed during naps (hrs/d)	3.5 ± 1.3	3.4 ± 1.1	3.6 ± 1.2	0.91
Sleep duration during naps	2.2 ± 0.7	1.9 ± 0.7	2.1 ± 1.0	0.22

Notes: Total time in bed is measured over a 24-hour cycle; Time in bed refers to the duration of a major sleep event; Sleep duration refers to the time spent sleeping during a major sleep event; Sleep *m*idpoint refers to the midpoint between bedtime and get up time.

### Sensitivity analyses

Our main results remained unchanged after excluding patients that were on sleep medication by the end of the treatment or after excluding patients that had less than 3 days of valid Emfit sleep data (Supplement S5).

## Discussion

This study provides a comprehensive examination of sleep patterns throughout the course of neoadjuvant chemotherapy treatment (i.e., over ~ 100 consecutive days with valid data). Our findings suggest that chemotherapy might affect time spent in bed, with patients experiencing more frequent sleep disturbances as treatment progresses. Interestingly, most changes in time in bed seem to occur by week 7, with sleep patterns stabilizing thereafter. Neoadjuvant chemotherapy did not seem to affect other dimensions of sleep. These results highlight the importance of developing interventions to mitigate the impact of neoadjuvant chemotherapy on sleep patterns.

Studies so far have relied on snapshots of sleep characterizations, typically measured either at the start or end of treatment or at the end/post-treatment. If we only had examined time spent in bed at the beginning and at the end of treatment, we would have concluded that sleep remained consistent throughout treatment. Instead, we observed that time spent in bed decreases during the initial four cycles of treatment. This finding is consistent with previous studies that used actigraphy to monitor sleep patterns, and suggests that chemotherapy treatment may lead to immediate changes in sleep once treatment starts^9,21^. A study by Ancoli-Israel et al.^9^ also found that sleep-wake cycle disturbances were more prevalent during the initial phase of treatment, with patients exhibiting more pronounced sleep disturbances at week 8 of chemotherapy compared to the sleep patterns observed at the beginning of treatment. Savard and colleagues also found that the first weeks of chemotherapy were associated with transient disruptions in sleep-wake rhythms and that repeated administrations of chemotherapy resulted in additional impairments in sleep-wake activity rhythms^21^. Disrupted sleep is a well-documented consequence of chemotherapy treatment and various organizations have issued recommendations emphasizing the importance of monitoring changes in sleep throughout cancer treatment^24^. In breast cancer patients, sleep disruption is linked to biological changes, such as circadian, immune, and metabolic alterations, and is associated with various side effects, comorbidities, and reduced quality of life, potentially affecting survival^25^. Our study suggests that treatment may impact some dimensions of sleep and highlights the importance of continuous sleep monitoring for capturing reductions in time in bed that occur during the first weeks of treatment.

We also observed that sleep quality as measured by reported sleep disturbances decreased over the course of treatment. These findings are consistent with Kreutz et al. that evaluated subjective sleep using the PSQI among women with breast cancer undergoing neoadjuvant chemotherapy. Similarly to our results, sleep disturbances, but not the remaining six dimensions of PSQI, were negatively affected by chemotherapy^6^. Our analysis of individual PSQI items related to sleep disturbances revealed a steady increase in sleep–wake disorders, a pattern closely associated with a diagnosis of insomnia^26^. One other study also observed that one of the most frequently reported reasons for sleep disturbances was waking up late at night or early in the morning (reported by over 50% of patients)^27^. In our study and by the end of treatment, ~ 70% of patients also reported similar disruptions in sleep. We also found that overall PSQI quality scores remained stable throughout treatment. This can potentially be explained by the poor sleep quality scores already prevalent at week 1 (mean score: 7.1). This is in alignment with the existing literature^22,28,29^ indicating that a breast cancer diagnosis can itself result in increased anxiety, and onset of depression^22^. For example, Ancoli-Israel and colleagues (2006) reported PSQI scores ~ 7.0 among patients awaiting initial cancer treatment. These findings highlight that sleep disturbances often occur at diagnosis and seem to persist throughout treatment^29^.

Our study suggests that cancer patients may use napping as a compensatory strategy for disrupted nighttime sleep patterns^8^. While napping can offer short-term benefits^30^ such as reducing inflammation and cortisol levels after sleep deprivation,^31^ excessive or poorly timed naps may negatively impact nighttime sleep quality and daytime function^32^. Cancer treatment and resulting side effects (e.g., fatigue)^20^ may contribute to longer naps in cancer patients, potentially reflecting the severity of sleep disturbances and the increased need for additional rest^33^. However, evidence suggests that late afternoon naps may interfere with nighttime sleep and potentially lead to poorer daytime function and reduced overall vitality^32^. Future research is needed to explore optimal napping strategies among breast cancer patients, while considering both the potential benefits and drawbacks of daytime napping.

Interestingly, we did not find sleep timing or day-to-day variations in sleep timing (i.e., sleep regularity) to be affected by treatment. About 30–50% of cancer survivors experience sleep disorders during cancer treatment and are at high risk for circadian disruption^10^. Circadian disruption refers to the misalignment of behavioral and environmental factors with physiological processes^34^ and can impact cancer prognosis^35^. In our study we quantified sleep midpoint variations as a potential marker of circadian changes^36^. Contrary to our hypothesis, treatment did not appear to impact sleep timing, suggesting that our patients were not at risk for major circadian disruption while in treatment. Our preliminary study did not include direct measures of circadian disruption and therefore more research is needed to develop more direct and refined measures of circadian disruption during cancer treatment.

Our study is not without limitations. Sleep data were collected using a device based on ballistocardiography, rather than polysomnography, the gold standard measure of sleep. However, polysomnography is better suited for laboratory-based sleep measurements and short duration (i.e., 2–3 days) characterizations of sleep, and hence not ideal for capturing usual sleep patterns over extended periods in real-world settings. Moreover, the Emfit device used in our study can only monitor sleep in a single location. In our study, patients were asked to install the Emfit device under their primary location/bed for nighttime sleep. It is possible that patients accumulated some sleep and naps in other locations^37^ and hence we were not able to capture those sleep events. Our measurements of sleep patterns were also imperfect. The Emfit is known to overestimate sleep duration and hence our absolute estimates for sleep duration need to be interpreted with caution^16,17^. There is also limited validity information on the Emfit accuracy for measuring other dimensions of sleep including timing and quality. Therefore, we focused on relative changes in sleep throughout the duration of treatment. Emfit sleep duration is moderately correlated with PSG (*r* = 0.5)^16^ and thus our trends over the course of treatment are still relatively accurate and not substantially affected by Emfit inaccuracies for absolute values of sleep duration. Agreement with PSG for sleep timing is also within the expected range (*r* = 0.64), while the agreement for sleep quality is poor (*r* = 0.26). The inaccuracies of Emfit for capturing sleep patterns are similar to those observed with actigraphy. Actigraphy estimates for sleep timing and quality (e.g., WASO) show correlations in the range of *r* = 0.48–0.76 for sleep timing and *r* = 0.37–0.58 for sleep quality, respectively, with concurrent estimates obtained from PSG^17,38^. We were also not able to capture pre-treatment sleep patterns, as patients are quickly scheduled for treatment once they have their first consultation at our hospital. Therefore, our representations of sleep are limited to changes in sleep occurring during treatment. Another limitation of our study is the small sample size, which may explain the high inter-individual variability, as indicated by the large standard deviations across all measures, resulting in lack of statistical power. Future studies with larger sample sizes are needed to confirm the trajectories of sleep patterns among cancer patients throughout treatment. Additionally, this study did not examine potential additional factors contributing to sleep disruptions, and future research should explore these causes to better understand chemotherapy-induced sleep disturbances. Lastly, the generalizability of our findings is limited to Caucasian, highly educated women, with high socioeconomic backgrounds, undergoing neoadjuvant chemotherapy. Therefore, the findings of our study may not be generalizable to women from different socio-economic backgrounds or receiving other forms of treatment. Thus, our extensive sleep monitoring protocol during a critical period of breast cancer treatment still provides novel insights on how chemotherapy may affect sleep patterns.

## Conclusions

Our study examined over 100 consecutive days of sensor based sleep and suggests that neoadjuvant chemotherapy may significantly impact sleep patterns in breast cancer patients. Sleep patterns appeared to be particularly affected during the initial seven weeks of treatment, with clear reductions in time spent in bed. Additionally, the prevalence of sleep disturbances, including insomnia symptoms, increased progressively over the course of treatment, while sleep timing and regularity appeared to remain stable. These findings highlight critical periods where breast cancer patients may be particularly vulnerable to sleep disruptions during chemotherapy. Increased recognition of these potential changes in sleep by healthcare professionals is essential as it may help set realistic expectations for patients, reducing anxiety, fear, and the overall impact of treatment. Future research should explore sleep interventions for improving sleep patterns during neoadjuvant chemotherapy and hence improve patients’ well-being and quality of life.

## Electronic supplementary material

Below is the link to the electronic supplementary material.

Supplementary Material 1

## Data Availability

Data is provided within the manuscript or supplementary information files. The datasets used during the current investigation are available from the corresponding author on reasonable request (carla.malveiro@fundacaochampalimaud.pt).
