# Supplementary material for: Sensor based sleep patterns and reported sleep quality in breast cancer patients undergoing neoadjuvant chemotherapy
Source: Sci Rep. 2025 Jul 11;15:25075. doi: 10.1038/s41598-025-99208-0 (PMC12254343; doi:10.1038/s41598-025-99208-0)
Supplement: Supplementary file 1 — Supplementary Material 1 [file 41598_2025_99208_MOESM1_ESM.docx]

**SUPPLEMENTARY INFORMATION**

**S1.** Study protocol of the NEO-Program (NCT05297773): A Randomized Controlled Trial of Supervised Exercise and Ki-67 among Breast Cancer Patients Undergoing Neoadjuvant Treatment.

**Project Summary**

The NEO study is comparing the effects of two distinct exercise types—Aerobic Training (AT) and Strength Training (ST)—against a stretching/relaxation control group (CG) on Ki-67 in breast cancer (BC) patients undergoing neoadjuvant treatment (hormone therapy or chemotherapy). The primary endpoint of this trial is Ki-67 - a marker of tumor proliferation - to assess the impact of exercise on tumor biology during the neoadjuvant treatment period.

The NEO study is ongoing and will include 132 physically inactive BC patients, aged ≥18 years and nonpregnant, divided into three groups: 1) AT (continuous cycling, treadmill, or rowing); 2) Strength Training (whole-body strength exercises); and 3) CG (stretching/relaxation). All participants will undergo their assigned interventions during the neoadjuvant therapy period (±4-6 months, between diagnosis and surgery). Exercise interventions are designed to ensure equal energy expenditure in both exercise groups, with a weekly target of 10 kcal/kg.

The trial will be conducted over a period of four years, and several secondary endpoints will be measured, including body composition, resting metabolic rate, physical fitness, quality of life, fatigue, depression, anxiety, physical activity levels, sedentary time, sleep quality, tumor biology, adverse events, lipid and glycemic profiles, inflammatory markers, and cardiovascular health.

This research will provide valuable insights into the potential of tailored exercise types to modify tumor biology and improve physical and psychological outcomes in BC patients undergoing neoadjuvant therapy.

**Rationale for NEO & background information**

Exercise performed during the neoadjuvant period can modulate several host- and tumor-related pathways and improve tumor gene expression,^1–3^ while also having beneficial effects on physical fitness and quality of life in these patients.^4–6^ However, most of the investigations describing the effects of exercise have been examined during post-surgery/adjuvant therapy or in cancer survivors, with only a few trials exploring the impact of exercise during the neoadjuvant period, i.e., treatment phase preceding surgery, namely in breast cancer^4^.

To address the lack of knowledge regarding the impact of different exercise modalities on breast cancer patients undergoing neoadjuvant treatment, a supervised exercise intervention was implemented. This 3-arm randomized controlled trial (RCT) aimed to evaluate and compare the effects of two distinct exercise regimens – aerobic and strength – against a stretching/relaxation control group on breast cancer patients undergoing neoadjuvant therapy. The primary outcome was tumor proliferation index, measured as KI67% assessed at both baseline and at the end of the intervention.

**Scientific objectives**

**Main goal**

- To determine and compare the effects of two distinct exercise regimens (aerobic and strength) against a stretching/relaxation control group on Ki-67 in breast cancer patients undergoing neoadjuvant treatment

**Secondary Goals**

- To determine and compare the effects of two distinct exercise regimens (aerobic and strength against a stretching/relaxation control group on:
  - Body composition (fat mass, fat-free mass, bone mineral density and content; waist and hip circumference)
  - Resting metabolic rate
  - Physical fitness (cardiorespiratory fitness and muscle strength)
  - Quality of life, fatigue, anxiety, and depression
  - Sleep quality
  - Physical activity levels and sedentary time
  - Tumor size, hypoxia and immune profile
  - Glycemic, lipid, and inflammatory profile

**Description of methods and materials**

**Study Design**: Participants are being enrolled in a randomized controlled trial (The Neoadjuvant Exercise Oncology Program – The NEO-Program) undertaken at Champalimaud Foundation. The trial is being conducted during the full neoadjuvant period (between diagnosis and breast surgery) and participants are being divided into three groups: 1) AT; 2) ST; 3) CG (relaxation/stretching exercises). There have been no changes to trial outcomes since the trial commenced.

**Recruitment**: Participant recruitment began in March 2021 and is expected to continue until December 2025 at the Champalimaud Clinical Center Breast Unit. The intervention is lasting 4-6 months per participant, depending on the type of neoadjuvant therapy.

- **Inclusion criteria**: histologically confirmed breast cancer (stage 0-III); scheduled for neoadjuvant therapy (cohort A – chemotherapy; cohort B – endocrine therapy); women aged ≥ 18 years; nonpregnant; not involved in any structured exercise program in the past 6 months; medical approval to participate in the study; willingness to attend exercise sessions.
- **Exclusion criteria**: Treated for any cancer in the past 5 years (except basal cancer or cervical cancer); uncontrolled heart disease (heart failure, uncontrolled coronary heart disease, uncontrolled hypertension and cardiovascular disease); diabetes mellitus; lung condition (chronic obstructive or restrictive pulmonary disease); psychological disorders (dementia, Alzheimer and Parkinson Disease); severe disability, or other medical condition that prevents from exercise training; alcohol or drugs abuse.

**Randomization and Allocation Concealment**

Participants are being randomly assigned to one of three intervention groups in a 1:1:1 allocation ratio: 1) AT; 2) ST; 3) CG, using a computer-generated random sequence created by an independent statistician. To ensure allocation concealment, participant assignments are conducted by a researcher uninvolved in recruitment or assessments. Given the nature of the interventions, blinding of participants and exercise professionals is not feasible; however, outcome assessors and data analysts will remain blinded to group allocation to minimize potential bias in evaluating primary and secondary outcomes. All intervention/protocol procedures that are not usually included in Champalimaud Foundation’s protocol, are being performed by professionals, researchers, and students from the Exercise and Health Laboratory.

**Sample power calculations**: With a calculated sample size of 22 participants per group (66/cohort, 132 total), already accounting for a 30% dropout, this study will have a statistical power of 80% to detect a 5.7% mean difference in Ki-67 between the exercise groups and control, while considering a type error I of 5% and a standard deviation of 7.3% for Ki-67 as previously documented in a chemoprevention trial^8^. The power and sample calculations were performed using a study that reported the effect of medication on KI-67 since no study has evaluated the effects of an exercise intervention on that variable and has obtained significant results. The same approach was used by Ligibel and colleagues^2^ in their exercise trial.

**Exercise intervention:** The intervention is designed to have equal energy expenditure throughout the exercise sessions in both exercise groups (weekly target of 10 kcal/kg). All exercise sessions are being supervised by exercise professionals and monitored with heart rate monitors.

- AT: participants will perform continuous cycling, treadmill, or rowing at 30%-35% of their heart rate reserve (HRR) for two weeks (phase 1: adaptive phase). Then, the intensity will increase every 2-3 weeks up to a maximum of 80% of HRR (vigorous intensity), depending on participant tolerance.
- ST: initially, and during the first two weeks of the adaptive phase, participants are performing two sets of 12-15 repetitions (40%-RM, rate of perceived exertion: 9-11) of 8 exercises for upper and lower body (leg press, leg extension, leg curl, dead lift/glute bridge, plank, dead bug, seated row, shoulder press). Then, the participants are performing 3 sets of each exercise (70%-RM, rate of perceived exertion: 14-15). Throughout the program, load is being adjusted to keep the perceived exertion of the participant at, at least, 14-15 and according to individual tolerance.
- CG: weekly stretching/relaxation session for 45 minutes.

**Measurements**

**Main outcome**

- **Ki-67**: Pathology evaluation while using initial tumor biopsy and surgical specimen.

**Secondary outcomes**

- **Height and weight**: electronic scale (before each training session).
- **Circumferences**: according to the standardized procedures defined by National Health Institute and World Health Organization (biweekly).
- **Fat mass, fat-free mass and bone mineral content/density**: Dual-energy X-ray absorptiometry (baseline, in the middle, and in the end of the intervention).
- **Resting metabolic rate**: indirect calorimetry using a gas analyzer (baseline and end of the intervention).
- **Cardiorespiratory fitness**: maximal cardiopulmonary exercise testing on a cycle ergometer while using indirect calorimetry (baseline and at the end of the intervention).
- **Muscle strength**: handgrip and mid-thigh pull test (baseline, in the middle, and at the end of the intervention).
- **Quality of life**: EORTC QLQ-C30 and EORTC QLQ-BR 23 (baseline, in the middle, and at the end of the intervention).
- **Fatigue**: FACIT scale (baseline, in the middle, and at the end of the intervention).
- **Depression and anxiety**: The Hospital Anxiety and Depression Scale (HADS) (baseline, in the middle, and at the end of the intervention).
- **Sleep quality**: Pittsburgh Sleep Quality Index (subjectively; baseline, in the middle of the intervention, at the end of the intervention) and ballistocardiography (EMFIT QS; objectively measured throughout the entire intervention).
- **Physical activity levels and sedentary time**: accelerometer (baseline, in the middle of the intervention, at the end of the intervention).
- **Tumor size and hypoxia**: resonance magnetic imaging (baseline and at the end of the intervention).
- **Immune profile**: tissue microarray blocks, with a sampling of the tumor-infiltrating lymphocytes hotspots from the initial biopsy and surgical specimen material, will be constructed. An immunohistochemical study with CD8+, CD4+, and CD56+ will be performed on the tissue microarray blocks (baseline and at the end of the intervention).
- **Glycemic and lipid**: blood samples (baseline, in the middle, and at the end of the intervention).
- **Inflammatory profile**: blood samples using flow cytometry with a Cytometric Bead Array technique and ELISA kits (baseline and at the end of the intervention).

**Distribution of key indicators at baseline**

So far, allocated arms are similar in height, but there are differences observed in other variables, at baseline. AT is younger (47.1 ± 7.0years), has a lower body weight (60.1 ± 4.7 kg), and a lower BMI (23.0 ± 1.3 kg/m²) when compared to ST group and CG. CG has the highest BMI (27.5 ± 4.2 kg/m²). VO₂max is higher in AT (25.4 ± 2.7 ml/min/kg), whereas ST and CG have nearly identical values (21.5 ± 5.2 and 21.4 ± 4.1 ml/min/kg, respectively), at baseline.

**Expected outcomes**

This innovative research project will address an important gap in exercise-oncology and improve our understanding of the direct effects of exercise on breast cancer biology and tumor progression in women with breast cancer. Moreover, the research design involving 3-arms makes it possible to compare AT and ST - the two most common types of exercise – against a CG, and, therefore, understand the different impacts of the two exercise stimuli on different outcomes, especially on tumor proliferation, quality of life, and therapy-related side-effects. We anticipate that exercise intervention will reduce Ki-67, improve body composition by decreasing fat mass and increasing fat-free mass, and help maintain bone mineral density in patients with BC undergoing NAT. AT will lead to greater fat loss and circumference reductions, while ST will promote higher fat-free mass gains. Exercise will increase Phase Angle, though it may temporarily drop after chemotherapy, with a less noticeable decline in exercise groups. It will improve physical fitness, with AT improving CRF and ST increasing muscle strength. Exercise will improve quality of life (EORTC-QLQ C30; EORTC-BR23), perceived fatigue (FACIT), depression and anxiety (HADS) and sleep quality (EMFIT, PSQI). It will also decrease tumor size, especially with AT, while improving oxygen delivery and tumor vascularization. Exercise will reduce the frequency and severity of adverse events, increase the pathologic complete response rate, and lower preoperative endocrine prognostic index (PEPI) and Residual Cancer Burden (RCB) scores. Moreover, the increase in CRP, TNF-α, IL-6, IL-8, IL-1β, IL-1ra, and IGF-1 and the decrease in BDNF, IL-12p70, IL-10, CD8+, CD4+, CD56+, oncostatin M are expected to be significantly smaller in exercise groups compared to the CG. Exercise will decrease fasting glucose, HbA1c, insulin, LDL, triglycerides, while increase HDL. It will also improve cardiac function by reducing cardiotoxicity, carotid intima-media thickness, and pulse wave velocity.

**References**

1. Jones, L. W. *et al.* Modulation of circulating angiogenic factors and tumor biology by aerobic training in breast cancer patients receiving neoadjuvant chemotherapy. *Cancer Prev. Res. Phila. Pa* **6**, 925–937 (2013).

2. Ligibel, J. A. *et al.* Impact of a Pre-Operative Exercise Intervention on Breast Cancer Proliferation and Gene Expression: Results from the Pre-Operative Health and Body (PreHAB) Study. *Clin. Cancer Res. Off. J. Am. Assoc. Cancer Res.* **25**, 5398–5406 (2019).

3. Rao, R. *et al.* Bootcamp during neoadjuvant chemotherapy for breast cancer: a randomized pilot trial. *Breast Cancer Basic Clin. Res.* **6**, 39–46 (2012).

4. Lee, K., Norris, M. K., Wang, E. & Dieli-Conwright, C. M. Effect of high-intensity interval training on patient-reported outcomes and physical function in women with breast cancer receiving anthracycline-based chemotherapy. *Support. Care Cancer Off. J. Multinatl. Assoc. Support. Care Cancer* **29**, 6863–6870 (2021).

5. Sturgeon, K. M. *et al.* Feasibility of a tailored home-based exercise intervention during neoadjuvant chemotherapy in breast cancer patients. *BMC Sports Sci. Med. Rehabil.* **14**, 31 (2022).

6. Hornsby, W. E. *et al.* Safety and efficacy of aerobic training in operable breast cancer patients receiving neoadjuvant chemotherapy: a phase II randomized trial. *Acta Oncol. Stockh. Swed.* **53**, 65–74 (2014).

7. Malveiro, C. *et al.* Effects of exercise training on cancer patients undergoing neoadjuvant treatment: A systematic review. *J. Sci. Med. Sport* **26**, 586–592 (2023).

8. Dowsett, M. *et al.* Effect of raloxifene on breast cancer cell Ki67 and apoptosis: a double-blind, placebo-controlled, randomized clinical trial in postmenopausal patients. *Cancer Epidemiol. Biomark. Prev. Publ. Am. Assoc. Cancer Res. Cosponsored Am. Soc. Prev. Oncol.* **10**, 961–966 (2001).

**S2.** Definitions for sleep metrics.

| Variable | Definition | Unit |  |
| --- | --- | --- | --- |
| Total time in bed | The total amount of time a person spends in bed, regardless of whether they are asleep or awake over the course of a 24-hour day | hrs/d |  |
| Time in bed | The total amount of time that a person spends in bed regardless of whether or not they are sleeping during that time | hrs/d |  |
| Sleep duration | The quantity of time that a person sleep | hrs/d |  |
| Wake after sleep onset (WASO) | amount of time a person spends awake after initially falling asleep, throughout the main sleep period | min |  |
| Sleep Efficiency | relationship between sleep duration and time in bed: (sleep duration/time in bed) x100 | % |  |
| Bedtime | Specific time a person goes to bed to begin their sleep episode | hh:mm |  |
| Get out of bed | Specific time a person gets out of bed after waking up, marking the end of their sleep episode | hh:mm |  |
| Sleep Midpoint | Midpoint between bedtime and get up time. | hh:mm |  |
| Sleep Regularity | Standard deviation of the sleep midpoint over recorded days with Emfit data. | Standard Deviations |  |
| % participants with 1+nap per day | Percent of participants with one or more naps during the day. It is calculated by dividing the number of participants with 1+ naps per day by the total number of participants and multiplying by 100. | % |  |
| Time in bed during naps | The total amount of time a person spends in bed, regardless of whether they are asleep or awake, during naps. | hrs/d |  |
| Sleep duration during naps | The quantity of time that a person sleeps during naps. | hrs/d |  |
| %, percent; d, day; hrs, hours; n, number; WASO, wake after sleep onset; hh:min, hours:minutes; Std, standard deviation | | | |

**S3.** Sleep Regularity throughout treatment.

| Treatment | Sleep Regularity |
| --- | --- |
| Weeks | **Mean ± Std** |
| 1 | 4.1 ± 1.5 |
| 2 | 5.2 ± 6.0 |
| 3 | 5.2 ± 7.2 |
| 4 | 5.1 ± 5.8 |
| 5 | 3.5 ± 1.4 |
| 6 | 4.1 ± 1.5 |
| 7 | 3.5 ± 1.4 |
| 8 | 4.2 ± 1.2 |
| 9 | 3.5 ± 1.2 |
| 10 | 3.5 ± 1.5 |
| 11 | 5.2 ± 7.1 |
| 12 | 5.0 ± 6.2 |
| 13 | 4.0 ± 1.3 |
| 14 | 5.2 ± 6.0 |
| 15 | 4.1 ± 1.3 |

**S4.** Percent participants with 1+ naps/day and average time in bed during naps.

| Treatment | Participants with 1+ naps/day | Time in bed during naps | Sleep Duration during naps |
| --- | --- | --- | --- |
| Weeks | **%** | **Mean ± Std** | **Mean ± Std** |
| 1 | 23.1 | 2.7 ± 0.3 | 2.0 ± 0.5 |
| 2 | 22.7 | 3.0 ± 0.5 | 2.0 ± 0.7 |
| 3 | 25.0 | 4.0 ± 1.3 | 1.7 ± 1.0 |
| 4 | 16.7 | 3.5 ± 0.9 | 2.2 ± 0.4 |
| 5 | 20.8 | 3.3 ± 0.5 | 2.1 ± 0.5 |
| 6 | 20.8 | 4.1 ± 2.0 | 2.9 ± 1.4 |
| 7 | 12.5 | 3.7 ± 1.7 | 2.1 ± 0.4 |
| 8 | 25.0 | 3.1 ± 1.1 | 1.9 ± 0.3 |
| 9 | 20.8 | 3.6 ± 0.9 | 1.9 ± 0.6 |
| 10 | 8.7 | 4.0 ± 2.6 | 2.5 ± 1.0 |
| 11 | 45.5 | 3.5 ± 1.3 | 1.8 ± 0.9 |
| 12 | 34.8 | 1.4 ± 2.4 | 2.2 ± 0.5 |
| 13 | 29.2 | 3.3 ± 1.1 | 2.1 ± 0.9 |
| 14 | 29.2 | 4.2 ± 1.6 | 2.6 ± 1.1 |
| 15 | 38.9 | 3.4 ± 1.4 | 2.2 ± 1.0 |

Percent participants with 1+ naps/day (p=0.30); Time in bed during naps (p=0.84); Sleep duration during naps (p=0.84).

**S5.** Sensitive analyses for Time in Bed by medication and compliance at week.

|  | Week 1 | Week 8 | Week 15 | β^1^ |
| --- | --- | --- | --- | --- |
|  | **Mean** ± **Std** | **Mean** ± **Std** | **Mean** ± **Std** |  |
| Full Sample (n=24) | 9.3 ± 1.4 | 8.7 ± 1.3 | 9.0 ± 1.4 | -0.11 |
| Without Sleep Medication (n=16) | 9.2 ± 1.5 | 8.6 ± 1.5 | 8.7 ± 1.5 | -0.07 |
| 3+ days/week of Emfit (n=21) | 9.2 ± 1.7 | 8.6 ± 1.3 | 8.9 ± 1.4 | -0.10 |
| Sleep Medication was derived from a question in the Pittsburgh Sleep Quality Index.  ^1^β is the coefficient obtained from the linear mixed models and indicates changes in time in bed per 1-week of chemotherapy treatment. | | | | |
